# Creating advantages through franchising in healthcare: a qualitative, multiple embedded case study on the role of the business format

**DOI:** 10.1186/s12913-014-0485-5

**Published:** 2014-11-02

**Authors:** Karlijn J Nijmeijer, Robbert Huijsman, Isabelle N Fabbricotti

**Affiliations:** Institute of Health Policy and Management, Erasmus University Rotterdam, P.O. BOX 1738, 3000 DR Rotterdam, The Netherlands; The Rotterdam Eye Hospital, P.O. BOX 70030, 3000 LM Rotterdam, The Netherlands

**Keywords:** Franchise, Business format, Professionals, Performance, Organization, Cooperation

## Abstract

**Background:**

Business format franchising is an organizational form that originates from the business sector. It is increasingly used in healthcare, being a promising organizational form for improving the competitiveness and efficiency of organizations, the quality of care, and the professional work environment. However, evidence is lacking concerning how these healthcare franchises should be designed to actually deliver the promised benefits. This study explores how the design of the central element in franchising, the business format (i.e., brand name, support systems, specification of the products and services), helps or hinders the achievement of positive results.

**Methods:**

A qualitative comparative embedded case study was conducted. The cases focused on three Dutch healthcare franchises providing mental healthcare, hospital care and care for the intellectually disabled. The data were collected through document analyses, observations, and 96 in-depth, semi-structured interviews with franchisors and unit actors (franchisees, unit managers, professionals). The interviews were recorded and transcribed verbatim. A conceptual model based on a systematic review of studies in other industries was used as an initial method for coding the data. New inductive codes were used to enrich and extend the analysis. The data were subjected to within-case and cross-case comparative thematic analyses.

**Results:**

Different business format designs have different effects on results, as perceived by franchisors and unit actors. The analysis revealed how this variation in perceived effects can be explained by different dynamics with regard to system-wide adaptation, local adaptation, professionals’ resistance to change, ease of knowledge sharing, bureaucracy, overhead, uniform brand presentation, accelerating effects and reliable performance levels. The analysis resulted in a new typology of four types of business formats, showing how combinations of business format elements facilitate or hinder the achievement of different types of results.

**Conclusions:**

Practitioners using healthcare franchising as a model to improve client-related, strategic, organizational and professional results should carefully consider how to design their business format in order to facilitate the achievement of desired results. The developed typology can be used as a starting point for these practitioners and as a basis for future scholarly research. Further quantitative research is recommended to confirm the results.

**Electronic supplementary material:**

The online version of this article (doi:10.1186/s12913-014-0485-5) contains supplementary material, which is available to authorized users.

## Background

The organizational model of franchising is increasingly applied by healthcare organizations to overcome challenges such as increasing competition [1], rising expenditures [2], deficiencies in quality of care [3], poor diffusion of innovations [4] and unsatisfied professionals [5]. Franchising originates from trade and industry and involves a contractual arrangement between two firms: the franchisor and the franchisee. The franchisee buys the right to provide health care services with the use of the franchisor’s business format [6]. The business format consists of a brand name, support systems, and specification of the products and services that need to be delivered [7,8]. Franchisees deliver services at locations close to clients while being supported with tried-and-tested practices, knowledge sharing facilities, and other operational and management support as described in the business format [9–11]. In some franchise systems, certain units are owned by the franchisor and operated by employed managers who use the same business format as franchisees. Currently, in the USA, there are at least 35 healthcare franchises in sectors such as elderly and home care, eye and hearing care, (para)medical care, and mental health care,^a^ 21 in the Netherlands, and 15 in both the UK^b^ and Canada.^c^ Fifty-three social healthcare franchises in various Asian and African countries were documented in 2011 [12].

Those who start operating a healthcare franchise expect it to be a successful model, either for commercial or social reasons. Two types of franchises are used in which actors partially have similar expectations. The first is a model with small-scale independent entrepreneurs (i.e., a stand-alone model) [13]. In developed countries, this type is often used as an alternative to large bureaucratic healthcare organizations. Actors expect that the combination of local entrepreneurship and support through the business format stimulates the professional satisfaction, efficiency and quality of care. This is thought to be achieved by restoring the autonomy of professionals in care provision while supporting them with effective practices and developed innovations [10,11]. Additionally, providers expect that the shared positioning with a brand name and clearly defined services may assist in the creation of competitive advantages [14,15]. In the second type of model, existing organizations become a franchisee for part of their care services (i.e., fractional model). Franchisees who choose this model also expect to improve their competitive position, quality of care, efficiency, financial performance and professional work environment. They expect to achieve these results through proven practices from the business format, the operational support [9], the shared branding [15], the possibilities for knowledge sharing and development [14,15], and the access to innovations originating from the franchisor and other franchisees [14].

However, difficulties may also arise. Franchising requires uniformity to achieve economies of scale and to build a strong brand name. This can reduce professionals’ autonomy and decrease their work satisfaction or the quality of care for customized services [11]. Moreover, professionals can misuse their powerful roles to resist the implementation of certain business format elements that are necessary to reach competitive advantage and efficiency but are not in the professionals’ interests [11]. Controlling the quality of services provided by professionals may also be difficult for the franchisor because he may lack the specialized knowledge to do so, yet the system’s reputation depends on the quality of services provided [11,14].

Although increasingly pursued, healthcare franchising seems to produce varying results. Both positive [9,16–19] and negative outcomes [9,17,20] have been found for clients, professionals and organizations in both developed and developing countries. Determining what accounts for these differing results is becoming increasingly important, as interest in franchising is growing. A recent systematic review showed that studies on this issue in healthcare are scarce [21]. However, studies in other industries have indicated that variations in the business format design between franchises explain the varying results across franchises. The business format influences results across franchises because it defines what support units receive [22,23], how much control they experience [24,25] and how strong the brand name is [22,25]. The question is whether these findings will apply to healthcare as well. We therefore empirically explore the following question: *How is the business format design perceived to facilitate or hinder the achievement of positive results with franchising, and why?*

### Objectives of the study

This study aims to contribute to the knowledge on how the organizational model of franchising can be effectively applied in healthcare. To this end, we qualitatively explore the views of franchisors and unit actors (franchisees, professionals, unit managers) regarding the help or hindrance of their business format design—a key element in franchising—in realizing strategic, organizational, professional and client-related results. We explore their experienced relationship between the design of business format elements and the achievement of results and their explanations for the perceived relationship. The analysis provides in-depth insight regarding which designs of a business format are likely to promote positive results and which other structural, strategic and behavioral decisions may have to be made in the franchise system to ensure that the desired results are achieved. We integrate our findings in a model that can be used as a starting point by franchise practitioners and as a basis for future research.

### Conceptual model to explore the role of the business format in healthcare franchises

A systematic analysis of studies that have investigated the influence of the business format in other sectors (see systematic review of [26], p. 8–10 for a detailed overview) have indicated that multiple business format elements affect the achievement of results. We used these insights to build a conceptual foundation for exploring the role of the business format in healthcare.

Insights from other sectors show that the design of two business format components influences the results achieved within franchises. The first is the ‘front’ of the business format, which constitutes elements related to the positioning toward customers [27]. The positioning toward customers comprises the brand name strength and the franchise concept, which includes the collection of the attributes of the products and services of the franchise and the presentation thereof [27]. This component determines the attractiveness of the business format to customers [27] and has been shown to be important for realizing positive strategic and organizational results for franchisors [25] and franchisees [28,29].

The second component is the ‘back’ of the business format and comprises the format facilitators. These are the operating and management structures that aim to ensure that unit actors deliver the services in their units as defined in the positioning component and to build a strong brand name [27]. Studies in other sectors have shown that the design of two types of facilitators influences results: support services and control systems. Support services include elements that assist unit actors in starting up and operating a unit (e.g., training). Control includes the specifications and limitations from the franchisor to ensure that unit actors behave as deemed necessary (e.g., rules on client contact).

Studies have indicated that design differences in the type, amount and quality of support services are a major reason for varying results across franchise systems. First, support services only positively affect results if they are of sufficient quality because low-quality support is less helpful [30]. Second, only those franchisors that provide appropriate types of support positively affect their own strategic and organizational results [23,31] and as well as the franchisees’ satisfaction and performance [28,32]. The types of support that are appropriate to franchisees partially depend on what the franchisees want or need. For example, Merrilees and Frazer [32] found that marketing support was appropriate to promote results for higher-performing franchisees, whereas training and operational management support were more appropriate for younger and lower-performing franchisees. Third, studies showed that results across franchises vary because of differences in the amount of support provided. While extensive support can benefit franchisee results [33], it can have negative effects for franchisors, supposedly because developing and providing extensive support is expensive and labor-intensive [34].

Studies have indicated that differences in control levels are another reason for varying results across franchises. The design of three control elements was found to affect results: the selection of franchisees, the level of standardization and the level of centralized decision-making. First, studies have shown that the use of ample information and assessment methods to select franchisees with the right attitudes and expectations reduces the likelihood of unsatisfied franchisees who will quickly abandon the franchise [35] and increases success [36]. However, such strict selection can hinder rapid growth in system size [37]. Second, studies have shown that greater use of standardized operating instructions has financial benefits for franchisees, presumably because these steer them toward the tried-and-tested practices of the franchisor and to act in the interests of the clients and the entire system [38,24]. However, standardization may only be beneficial in the first contract years. In later years, franchisees’ adaptation to the local context may be more beneficial [8]. For franchisors, greater standardization may also have negative effects, as it can undermine the franchisees’ innovation efforts [25]. Third, more centralization may be positive for a franchisor [39], while franchisees may benefit more when decision-making is more decentralized [38,40] because it reduces opportunistic behavior [38].

## Methods

### Research design

We conducted a qualitative multiple embedded case study. Several cases were investigated at several levels of analysis [41]. The levels of analysis included both the franchisor level and the unit level, as research has shown that studying both is necessary to fully understand the processes and results created within franchise systems [42]. Qualitative research enabled us to explore these franchises in-depth and build a theory of *how* and *why* different business format elements are perceived to affect the achievement of results in a rarely investigated organizational form in healthcare – a franchise. The use of three franchise systems in three different healthcare sectors enabled us to confirm our findings (replication) and to identify diverging patterns across settings, thereby reaching more explanatory power and generalizability [41,43,44]. Within-case comparisons further enhanced the validity [41]. A conceptual model was used to focus attention on particular themes, to achieve a deeper analysis of an unexplored phenomenon and to extend theory [41]. Our study adheres to the RATS guidelines for qualitative research.

### Research setting

We conducted our study in the Netherlands, where approximately 21 healthcare franchises exist. Depending on the service provided, the franchises are reimbursed through (compulsory social) health insurance or private payments. Franchises providing hospital care, in-patient mental health care and in-patient long-term care for the disabled, youth and elderly are prohibited from working for-profit under Dutch law. Franchises providing other types of care officially can work for-profit, but many of them still do not have profit-making as their ultimate or only goal. Franchising is often used to improve quality, costs, and the work environment of care professionals.

Our cases were theoretically sampled as is recommended for a multiple case study [45]. First, we chose franchises providing different types of healthcare because scholars have theoretically assumed that this difference may play a role in how the business format is designed, the behavior of actors and the experienced results [11]. Second, we selected systems with existing organizations as franchisees (fractional model) and systems that started-up with individual entrepreneurs (stand-alone model) because it has been hypothesized that the existing work methods, culture and involvement of a larger-scale organization in a fractional model may lead to different requirements and effects of support and control in the business format [15]. We only used cases that had operated for at least three years and that were willing to share their sensitive insights. The selected franchises provide mental healthcare (system 1), hospital eye-care (system 2) and care for the intellectually disabled (system 3). A description of the cases regarding the elements of interest in this study is provided in Table 1. As shown in this table, system 1 and 2 both started franchising to obtain a stronger positioning in a market that became increasingly competitive as a consequence of changing policies and regulations by the Dutch government. They also aimed to improve the quality and efficiency of care in existing organizations, both for idealistic and competitively instrumental reasons. Under Dutch law, both systems are obliged to work not-for-profit. System 3 was founded to provide a qualitatively better alternative to regular care for the intellectually disabled. The system officially is a for-profit system.

**Table 1:** **Description of the cases**

	**System 1**	**System 2**	**System 3**
***Background information***			
Service	Mental healthcare	Hospital care (eye-care)	Care for the intellectually disabled
Year of establishment	2004	Franchise since 2007, system started in 2003	2003
Motive for franchising	Gain stronger position in more competitive market through high-quality, efficient care	Gain stronger position through provision of high-quality, efficient care in increasingly competitive market	Founded by a father who was highly dissatisfied with the quality of regular care for his intellectually disabled son
Type of franchise	Fractional: a portion of the care delivery of mental healthcare organizations is franchised.	Fractional: eye care departments of general hospitals are franchised.	Stand-alone: two care professionals operate a small-scale full-time living facility.
Number of units	26, owned by 4 franchisees. Units are daily operated by employed managers.	14, of which 11 franchised and 3 owned by the franchisor	107, of which 99 franchised and 8 owned by the franchisor
Payment method of care provided in units	(Obligatory) health insurance reimbursement, complemented with personal contribution of clients.	(Obligatory) health insurance reimbursement, complemented with personal contribution of clients	Personal budget of clients provided by governmental regional care offices following the Exceptional Medical Expenses Act
Contractual payments	All franchisees are shareholder of the franchise. All costs are proportionally divided and paid.	Fixed initial fee for quick scan/research before joining franchise. Ongoing annual fee comprising fixed base fee + variable fee per FTE ophthalmologist.	Fixed initial fee and fixed annual ongoing fee.
***Business format: positioning***			
Positioning toward customers	Specialized evidence-based ambulatory care provision to adults with an optimistic approach visible through office-like interiors, a specialized focus and excellent accessibility	Providing the entire spectrum of ophthalmology care in an excellent manner through regional and national cooperation, competent people, hospitable attitude, modern and smooth operations, and fine communication.	Providing care and living in a small-scale beautiful house with family-like atmosphere where disabled individuals can live as normal a life as possible with ample opportunities to do pleasant activities and receive love and attention
***Business format: support services***			
Support services provided to units	Branding, logo, website, folders, intranet, shared access system, operations manual (process improvement), routine outcome measurement (measure client progress), benchmarking, training, knowledge sharing/development structures	Branding, logo, intranet, website, publicity, frequent advisory support of franchisor representative to implement the operations manual with many ideas about process improvement, benchmarking, training, possibilities for shared purchasing, structures for knowledge sharing/development	Branding, logo, intranet, website, other publicity, facilitation of care building, facilitation of a loan, administration system, benchmarking, initial training, advisory support/coaching, lobby government, structures for knowledge sharing/development
***Business format: level of control***			
Initial control	Low	Low to medium	Medium, initially low
Level of standardized operating instructions in the franchise	• Care processes: medium to high	• Care processes: low, moving to medium	• Care processes: low to medium
	o fixed treatment programs, standardized intake (became looser), standardized pathways in treatment programs	o Protocols of professional bodies; currently works on certification of care pathways (e.g., which treatments, control moments)	o standardization of some boundary conditions: no. of customers allowed; guidelines about day-time care, medication lists, fixation
	• Non-care processes: medium to high (became looser)	• Non-care processes: low, tries to move to medium	• Non-care processes: medium
Level of centralized decision-making	• Care: now low on franchisee level (four franchisees are together franchisor), was more centralized at start), low-medium centralized from unit perspective	• Care: low	• Care: low
	• Non-care: now medium centralized from unit perspective; level differs per franchisee.	• Non-care: relatively low (almost all aspects that impact the franchisees are decided in consultation or by the hospital)	• Non-care: medium

### Data collection

The data were collected through semi-structured interviews, observations, and document analyses (see Additional file 1 for detailed information). These methods were used complementarily and improved validity through data triangulation [41,43]. Interviews were appropriate for gathering rich data about the actual design of the business format elements and the dynamics underlying their effects, as we could ask for experiences, perceptions and feelings [45]. To limit bias and acquire a representative overview, we purposively selected our interviewees based on characteristics that were shown to affect behavior and experienced results in prior research. Franchisor representatives with different functions were selected, as were units with varying ages (experience in the system) and operating in different geographical regions. Within each of the units, respondents with different functions were interviewed. All of the units and individuals who were selected for an interview were willing to participate; thus, selection bias is unlikely. A total of 96 interviews with 87 respondents (24 franchisor representatives, 37 professionals, 55 franchisee representatives, 14 company-owned managers; some respondents had two roles) were conducted between 2009 and 2012. Some respondents were interviewed more than once to obtain additional information and to check for developments over time.

A predetermined topic list based on the conceptual model was used during the interviews to increase the reliability and validity (see Table 2). The interviews lasted 1.5 hours on average and were recorded and transcribed verbatim. Documents were analyzed to prepare the interviews and to complement and triangulate the interview findings. Observations of meetings and daily practice were used to stimulate new lines of inquiry, to triangulate, and to obtain additional insights by observing the effects of the control and support elements in practice (e.g., discussion about the benefits of more control). During the observations and document analyses, the topic list from the interviews was used to ensure consistency.

**Table 2:** **Topic list used in the interviews, observations, and document analyses**

1)	experienced results of franchising;
2)	perceived contribution of their business format design, and more specifically: a) franchise concept, b) the brand name, c) perceived quality, type, amount of support, d) level of control (selection, standardization, decision-making rights), and – if relevant – the reason for choosing these designs;
3)	dynamics that result from these designs that explain the perceived effect;
4)	other aspects that lead to differing results despite using the same business format.

This study took a managerial and organizational perspective. Neither ethical approval of an Institutional Review Board nor written informed consent was required for this study according to the Dutch relevant legislation (law on medical scientific research with people (WMO), formal criteria Erasmus Medical Ethical committee^d^) because no medical data were used and patients were not involved in any way.

### Data analysis

The data were thematically analyzed. Themes were derived both deductively, using the theory from other industries, and inductively. We started by connecting deductively derived codes to the data. We subsequently refined the analyses by inductively applying new codes. We then used within-case comparison techniques to enrich and deepen the analyses. The consequent analysis was written down per case in a case report that was member-checked with case representatives to verify, adapt and complement the analysis. We then searched for consistent and distinct patterns among the three cases to further develop the analyses [46,47]. To verify and complement the analyses, the results of the between-case comparisons were member-checked in an advisory board meeting with representatives of all of the cases.

## Results

We first describe how the actors in the case study systems perceive their business format design to affect their achievement of positive results with franchising overall. We then analyze how actors feel that their pursued positioning, support services and level of control contribute to these experienced effects, as well as what dynamics explain these perceived effects.

### The experienced effects of the business format design in healthcare franchising

Consistent with differences in the design of the business format of the three case study systems, actors across systems differ in their perceptions of how their business format contributes to the achievement of positive results with franchising. Generally, the actors feel that their business format mainly either stimulates the achievement of positive results or has no impact. Only some perceive that the design of their business format hinders their financial performance, as well as the quality of care and work satisfaction in one system (see Table 3).

**Table 3:** Perceived influence of the business format on the achievement of various results within franchises over time*

**Franchise system**	**Actor**	**Competitive advantage**	**Financial performance & efficiency**	**Survival**	**Growth**	**Quality of care**	**Work satisfaction**
1	Franchisee and manager	+/0	+/0/-	+/0	+	+/0/-	+/0/-
	Professional	+/0	+/0	0	n.a.	+/0/-	+/0/-
2	Franchisor	+	First years: −, now: +	+	+	+	n.a.
	Franchisee and manager	+/0	+/0/-	+/0	n.a.	+	+/0
	Professional	+/0	+/0	+/0	n.a.	+	+/0
3	Franchisor	+	First years: −, now: +	+	+	+	n.a.
	Franchisee and manager	+	+/0/-	+	n.a.	+	+/0
	Professional	0	0	0	n.a.	+	+/0

*see Table 1 for a description of the design of the business format of each of the cases.

+ = perceived as facilitating to achieve this result type, − = perceived as hindering to achieve this result type, 0 = no perceived effect on the achievement of this result type. In determining the score, the focus was on shared agreements and disagreements. When respondents within the same actor-group varied in their opinion or when all respondents reported both positive and negative effects, a +/− sign was assigned.

As can be expected, franchisors primarily perceive positive effects of their business format design in achieving the types of results important to them: growth, competitive advantage, survival, quality of care, and financial performance after the initial years of establishment. Franchisees perceive stimulating effects as well, but they also see more negative or lack of effects. The franchisees in system 3 – who, in contrast to the other systems, all started a new unit with the use of the business format – are the most positive overall. Except for work satisfaction, for which some experience no effect, and for financial performance, for which some observe no or a hindering effect, the franchisees report experiencing only stimulating effects of their business format in achieving positive results. Some of the franchisees of system 2 – who engage the business format as an “additional tool” in their organization – also do not see a contribution to their competitive advantage and survival. The same applies to system 1, but here, some also perceive a hindering effect on achieving a high quality and work satisfaction, mainly during the initial years of the establishment of the franchise. Unit managers and professionals primarily perceive similar effects as the franchisees. This is not surprising as they work with the same business format and many professionals also fulfill a role as franchisee or unit manager. Therefore, we use the term ‘unit actor’ to commonly refer to franchisees, unit managers and professionals, when a finding applies to all of them.

### Positioning toward customers: perceived influences on results

How franchises position themselves toward customers is perceived to play an important part in the results of franchising. Specifically, the brand name strength and the concept play significant roles.

#### Strength of the brand

The respondents consistently reported that a strong brand name is advantageous, particularly through providing a strong position toward health insurers and the (local) government. A strong brand name legitimizes the preservation of financing for the franchised care, which basically increases the likelihood of survival, positive financial performance for units, and competitive advantages. Nevertheless, a strong brand name provides no guarantee for financing in a regulated healthcare environment with changing policies and regulations. A strong brand name is also believed to stimulate the quality of care and the work satisfaction because it provides a sense of belonging and additional motivation to perform well: *“It is nice to belong to something, you carry out a message together, and you have a strong brand name behind you that you need to keep strong together, work hard to keep that brand name strong, that nothing happens that will harm the brand.” (franchisee system 3).* Consequently, when a brand name is strong (as in system 3), it is regarded as a valuable resource and as a major reason for purchasing a franchise. When a brand name is not yet sufficiently strong in the perceptions of the unit actors (as in system 2), it is felt that the business format could offer more advantages. The actors initially expected that they would also gain by attracting more clients with their brand. However, this assumption appeared not entirely true for systems 1 and 2 due to production limits forced by insurers and the government to contain costs and because healthcare insurers continued purchasing care of all healthcare providers in the hospital and mental healthcare sector rather than purchasing larger volumes of care of only a selection of better-performing healthcare providers. As a franchisee of system 1 puts it, *“We designed [our franchise formula] based on content but also particularly on strong logistics and service because we were convinced that patients will vote with their feet when we deliver high quality care and that the market will come to us, as is normal in a market where you do a better job than others. But we do not have a normal market.”* Despite the advantages of branding, franchisees also consider branding as a risk because the mistakes of others can also affect them. However, no respondent considers this risk to outweigh the advantages.

#### Concept

Consistent with the theoretical framework, documents and interviews highlighted the importance of a concept including health care services that are valued by clients, purchasers (e.g., insurers), and referring providers. It seems that clients are attracted to a franchise because they like the concept, not particularly because it is a franchise. In system 1, respondents report that many clients found the optimistic approach, specialized treatment programs and office-like interiors to be attractive, and clients of system 3 were said to value the idea of a small-scale family-like atmosphere in a nice house where clients live as normal a life as possible. However, the cases show that governmental and political influences in healthcare can lower the demand or viability of the franchise, even if clients value the concept; system 1 received lower demand after the introduction of a financial contribution for clients, intended budget cuts for care for the intellectually disabled would have resulted in the discontinuance of new units, and production limits in hospital care reduced the possibilities of helping more patients. The concept also partly determines whether (potential) unit actors find the franchise attractive. The franchisees and a part of the professionals of system 1 liked the idea of specialized evidence-based care provision, while others disliked this idea and resisted changing their work methods; some even left. Hospitals and doctors found the idea of preserving the entire spectrum of eye-care and striving for excellence attractive. Many unit actors in system 3 were attracted to the idea of providing care in an autonomous unit after being disappointed in regular institutions.

Thus, the positioning component of the business format plays an important role in how franchises are valued. However, the support services and control systems in the business format should support realizing this positioning in practice. Therefore, the remainder of this section focuses on how support and control affect the achievement of positive results.

### Support services: perceived influences on results

Interviews, documents and observations consistently indicated that appropriate support services in regard to quality, type and amount facilitate the achievement of positive results with franchising. What exactly is appropriate partially differs for different types of results, for unit actors with different attitudes, skills and ages, for different types of franchise (fractional or stand-alone) and healthcare services, and for different perceived levels of contractual payments. We elaborate on these findings below.

A certain level of support services is perceived to positively relate to strategic, organizational, professional and client-related results for several reasons. First, for the franchisor, the provision of such support helps them to grow, create competitive advantages and survive by retaining and attracting unit actors to the system. Second, for franchisees, the support involves less risk and a stronger positioning in an environment of increasing competition and budget cuts from the government and insurers. Particularly for those franchisees that start up a new unit (system 3), the support also decreases the resources needed: *“If you do it by yourself (…)a) you need the financial resources, and b) you are far more vulnerable and you need to find out everything by yourself. And when something happens, you have nothing to rely on; you have no back-up.”* Third, unit actors feel that such support helps them to deliver high-quality, efficient care with less burden to themselves because they have to spend less resources in determining appropriate work methods: *“We could learn our lessons, we knew where we had to go, and we had the tools to do it.” (professional system 1).* Moreover, when ample support in non-care related activities is provided by the franchise, unit actors have to spend less resources in execution. As a franchisee of system 3 describes it, *“I do not have to spend too much time with non-care-related tasks, such as quality policies, but I can spend ample time providing care. I find that I now already spend too much time doing administration (…) Imagine the time that is spent when you have to do it all by yourself.”* Fourth, the cases show that the knowledge and experiences embedded in the support services accelerate improvement and implementation in local units because it is not necessary to *“reinvent the wheel everywhere”* and it persuasively shows the unit actors better working methods by peers. Fifth, the support steers the behavior of unit actors toward desirable performance levels and a uniform presentation, which is perceived to help stimulate quality of care, survival and competitive advantage.

However, from the perspective of both the franchisors and unit actors, the amount of support should not be too large. First, for the franchisor, extensive support requires a great deal of overhead, rendering the franchise relatively inefficient and expensive. As the franchisor of system 3 puts it, *“We need to remain efficient; we want to keep our overhead at 4.5%. So that implies that you must also dare to let it go.”* When the franchise system ages, more support can be provided with the same level of overhead, owing to greater experience and more developed services. According to the franchisor of system 2: *“We notice that over time our investments in new franchisees become lower. You can do it much more efficiently. You have much higher standards and ready-to-use material.”* Second, excessive support can hinder the quality of care, efficiency, competitive advantage, and work satisfaction in situations where local adaptation is important, such as in services that are complex and/or require customization and in units in which the franchised practices are added to existing and non-franchised practices (fractional model). The support then contains non-feasible and non-valuable practices for local units. As a franchisee of system 2 explains: *“By adding your own input, you keep it with your own culture, your philosophy, what fits within your own [unit].”* Third, extensive support can lead to the resistance and dissatisfaction of unit actors, particularly when it is presented as fixed and obligatory in a professionalized healthcare system like system 1. Unit actors then experience the support as a set of external work methods that they did not invent or own, feel violated in their autonomy, and feel that their opinions and capacities are not taken seriously. As several professionals of system 1 describe: *“The question was, do you unthinkingly copy it. In other words, you do not have to think anymore; you can do it this way, while people also felt the need to participate in thinking about the developments,”* and *“Who says that they know it better?”* Ultimately, the desirable level of support depends on the extent to which unit actors want to bring in their own ideas. As a franchisee of system 2 stated: *“You have the feeling that it is partially something of your own because you have collaborated on developing it.”*

The positive effects of support are experienced only when the support is perceived to be of the appropriate type and quality, while the negative effects are aggravated when the type and quality is perceived to be inappropriate. Some support types are generally perceived to be helpful in achieving positive results across all systems: the operations manual, performance measurement and benchmarking, a franchisor representative (account manager), and support that aids in profiling for clients, insurers and the government in an increasingly competitive and complex environment (e.g., via website, lobby, and folders). For other support types, the perceived appropriateness differs for the two types of franchise organizations: whereas support in process improvement was valued by existing organizations that have become franchisees (system 1 and 2, fractional type), support in building rent, a bank loan, and a shared administration system was felt to be most important by franchisees that started up as a new unit (system 3, stand-alone type). The appropriate type, quality and amount of support also differs across unit actors within the same system. Actors that differ in their performance levels, length of stay, and skills and attitudes have varying needs. Starting and less skilled unit actors primarily find support in starting up their units or implementing the formula helpful for realizing positive results. Older and higher-performing ones are more interested in marketing, profiling, maintenance, and continuous knowledge exchange, and also desire a lower amount of support: *“I know that I can call them when I need them. And that is enough. You can do it more by yourself.” (franchisee system 3).* In addition, some unit actors find it sufficient to share knowledge through the operations manual, the franchisor account managers, and professional scientific bodies. Others find facilitation of knowledge sharing meetings one of the most important tools in making a franchise cooperation valuable to professional healthcare organizations: *“Those program councils with [professionals], they are very stimulating groups. People really like to participate in that. It is a very important medium of knowledge exchange for us. That is the place where it all happens.” (franchisee system 1).* Unit actors also differ in whether they find training to be a valuable support type. Given these differences in the evaluation of support types, unit actors within the same system cannot all be satisfied in the same ways.

Franchisors and franchisees also evaluate the ultimate effects of the support services in relation to the level of contractual payments. From both the franchisor and franchisee perspectives, the contractual payments must enable the franchisor to provide high-quality support, develop improvement and innovations to maintain the value and competitiveness of the support services, and ensure brand recognition. When the franchisees perceive that they obtain inappropriate support in return for the level of contractual payments paid, they evaluate the overall contribution of the business format to their satisfaction and financial performance negatively. As the use and valuation of support services varies across unit actors within the same franchise system as a consequence of the variation in age, performance, attitudes and skills, so does the perceived reasonableness of contractual payments. In all of the systems, some of the franchisees express doubt about the worth of their payments, *“It is a lot of money that you pay, if you use that money yourself you can also accomplish much things”,* and fees have been a frequent topic of discussion. This discussion also regularly includes a discussion about the ‘ethical’ nature of asking fees in a societal sector like healthcare. Others within the same system feel that their payments are reasonable and are ultimately paid back by the efficiency gains of the support obtained, reducing the costs of healthcare overall, *“When you see our turnover and you can use the (…) support. If you see what they have achieved in those few years (…) In relation to that, you can be satisfied. And that you have to pay for it…if I were to try to achieve that by myself, I would not have succeeded.”*

### Level of control: perceived influences on results

Largely in line with our theoretical framework, our analysis shows that a certain level of control helps to ensure that actors deliver services as defined by the positioning component, either through the selection of new franchisees or through standardized instructions and centralized decision-making. Furthermore, our respondents in healthcare highlighted the importance of a certain level of monitoring and enforcement in this endeavor. However, excessive control appears to have a hindering effect on the achievement of positive results with franchising. Several process dynamics explain why both very high and very low levels of control are thought to have a negative effect. The most appropriate level of control seems partially situation-dependent. We elaborate on these findings below.

#### Control through the selection of new franchisees

Actors in all of the systems feel that a stricter selection of potential franchisees – either via a strict procedure or via self-selection through providing extensive information about the franchise – stimulates the achievement of a strong competitive position and high client satisfaction. It is thought to do so because consistently high performance levels can be better guaranteed. As the franchisor of system 3 describes: *“I think that you have to set high standards for which franchisees you want to include in your system, and even then it sometimes can go wrong with a franchisee (…) You want to have as much success as possible for your [clients].”* Through enlarging the chance of having suitable franchisees with the right expectations, stricter selection is also felt to stimulate franchisee satisfaction. As less satisfied and lower performing franchisees require greater time investments from the franchisor, stricter selection also stimulates the efficiency of support provision. However, the beneficial effects of strict selection can contrast with achieving competitive and efficiency advantages through system growth, a goal that benefits from less control. Like a franchisee of system 3 illustrates: *“At one moment, the speed of growth…with 20 new locations a year, you need to have 20 franchisees. Tensions arise. And then you actually select franchisees too easily; you select people that you regret in retrospect.”* An initial focus on growth can then complicate the achievement of consistent quality levels and a uniform formula implementation. As the franchisor of system 2 stated: *“When it is a franchise that everybody can join, what does it say then, that you are a member of the franchise? Because we want to position ourselves as a network that provides excellent care, and you say that all locations provide that excellent care.”*

#### Control through standardized operating instructions and centralized decision-making

The case study systems differ in their level of standardized operating instructions for care and non-care activities as well as in the level of centralized decision-making (see Table 1). The franchisors and unit actors from these varying systems reach a consistent conclusion: both extensively high and low levels of standardization and centralized decision-making are disadvantageous, although the optimal level differs across systems and result types. As our analysis indicates that the process dynamics underlying the role of standardization and centralized decision-making are largely similar, we describe the role of these two control elements in one section.

A certain level of standardization and centralized decision-making of both care and non-care processes is thought to facilitate the achievement of competitive advantages, survival and quality of care by steering the behavior of unit actors toward desirable, solid performance levels and uniform presentation throughout the system: *“Franchisees also say, I’d love to know what we must do and arrange at minimum, that my [unit] is OK, but that of my neighbor-franchisee is OK as well, because when something goes wrong there I have a problem too, we work with the same brand name.” (franchisor system 3).* Therefore, unit actors request more control when they perceive it as being too low, like in system 2: *“You want to guarantee that when I arrive at a [franchise] location, I should get the same service, the same access, the same quality of care; you have to decide upon that with each other. It cannot be that you come to one place…. (…) This is what we want to guarantee; this is what we deliver.”* Such a uniform presentation and predictable performance is felt to be particularly important when the franchise positions itself toward customers with predictable, efficient services, as in system 1, or when it is the desire of purchasers. More standardization and centralized decision-making also help stimulate the quality of care, work satisfaction, efficiency, competitive advantage, and survival by reducing the resources unit actors have to spend on choosing and applying appropriate and innovative work methods, allowing a stronger focus on the actual care provision. As the franchisor of system 3 stated: *“As a franchisor we are very good in deciding and organizing everything around the unit and [franchisee], to make their unit work. And we select the franchisees on their suitability for care provision, in that respect they can do whatever they want.”* Additionally, standardization and centralized decision-making stimulate results because they ensure a shared basis between units that makes system-wide adaptation, knowledge sharing, and performance monitoring relatively easier and more efficient.

However, the level of standardization and centralized decision-making should not be too high. For franchisors, extensive control leads to inefficiencies on the system-level as a consequence of the overhead and bureaucracy required. Moreover, it can harm growth because many professionals do not feel attracted to systems that they perceive as leaving little room for their own ideas, even if this feeling does not reflect reality, as in system 2: *“You give away a part of your right to say, and our physicians wanted to stay independent. They do not like the [franchisor] telling them what to do. The question of whether the franchise would improve our care delivery has hardly been a topic of discussion. It was quite easy: our physicians wanted to keep their autonomy and independence.” (potential franchisee in a newspaper).* On the unit level, extensive standardization and centralized decision-making restricts local adaptation. This hinders creating local competitive advantages, quality of care, efficiency and work satisfaction when customization to the customers’ needs is pivotal, according to the positioning component in the business format or the characteristics of the healthcare service: *“It is so personal what happens; care is such an individual, personal thing that you really need to have freedom to have an impact” (franchisor system 3).* Excessive franchisor control also hinders achieving these positive result types when local adaptation of the franchised practices to the characteristics of the local unit is important, such as in units that offer both franchised and non-franchised services (fractional franchise type) or units that differ in size. The franchise then becomes an inefficient bureaucracy that wastes staff and money in developing and implementing products that are not valuable and feasible in the local context, like in the initial years in system 1: *“They (…) said that everything needed to be done exactly as they did it in the [original] unit. But I cannot implement the same row of care pathways as they have there when I do not have as many care professionals as they have. That is impossible.”* These characteristics can also lead to dissatisfaction among unit actors and resistance to change among professionals because they may feel that they have insufficient autonomy, that their ideas and expertise are unimportant or not taken seriously, and/or that they are no longer owners of their work. Such a situational misfit ultimately reduces the actual steering possibilities and uniformity of the formula.

In all, the findings suggest that higher levels of centralized decision-making and standardization are advantageous for topics that apply to the entire system and are thus more efficient to arrange centrally, as well as for those topics that are important for a uniform image or to realize the intended positioning toward customers. The levels should be lower for topics that require local adaptation to fulfill the customers’, professionals’ and local unit’s needs.

#### Control through monitoring and enforcement

All our case study systems monitor the quality and/or financial performance of units to reveal whether they perform as intended (e.g., via audits, benchmarking, measuring client satisfaction and waiting times). Both franchisors and unit actors argue that such monitoring is always important for results because it provides valuable opportunities for learning and steering. However, particularly when support and control levels are lower, monitoring is felt to be important for competitive advantage, financial performance and the survival of the franchise because the monitoring results then provide an opportunity to show the attributes and performance of the franchise to the outside world.

Once the monitoring instruments identify a gap, not all systems can easily force adequate performance or the use of standards across units; system 1 and 2 have relatively low possibilities of doing this, and system 3 has some possibility. The franchisor of system 2 argues that their low ability in this regard makes it harder to create competitive advantages and to quickly improve the quality and efficiency in the units, *“There are no sanctions when a [franchisee] does not want to do something, then we say ‘it’s a pity that you have not achieved that goal’, but it has no consequences. (…) Learning from business, you can do more with that.”* Respondents feel that the healthcare culture, in combination with the involvement of professionals, makes it harder to employ such a hard franchisor-franchisee relationship. However, various actors argue that even if there are possibilities to force in the context of less professionalized and complex healthcare services, persuasion is a better choice. As the franchisor of system 3 stated: *“When a franchisee goes beyond what we find acceptable, then you have to be able as a franchisor to have a conversation, to talk about the real purpose of providing care within this formula, so that the franchisee says ‘oh yes, I have been so stupid’. If you succeed in that, that is much more valuable than just saying ‘this is not what we are doing here’ and withdraw his contract.”*

## Discussion

This paper aimed to explore how franchisors and unit actors perceive the design of the business format to affect the achievement of strategic, organizational, professional and client-related results with franchising, and to identify the reasons for these effects. The study shows that a strong positioning toward customers (clients, insurers, referring providers) helps to achieve positive results. However, whether this positioning is realized as intended—and the positive results thus achieved—is perceived to be influenced by the design of the support and control systems. Differences in design have different perceived effects. In regard to support, the amount, the quality and the type of the support are perceived to influence results. In regard to control, results are perceived to be influenced by the manner in which new actors are selected, the level of standardization and centralized decision-making, the extent of monitoring, and the ability of the franchisor to force the use of standards and adequate performance. We identified various process dynamics that are responsible for these effects. Different support designs are perceived to lead to differences in the level of overhead, resistance, local adaptation, and the extent to which the presentation is uniform, the performance of units is predictable, and innovation and implementation are accelerated. These differences are perceived to subsequently influence results. Similar reasons are perceived to underlie the diverging effects of different control designs. Variations in the level of bureaucracy, ease of knowledge sharing, and system-wide adaptation appear to be additional reasons. The combination of the two dimensions ‘level of control’ and ‘extent of support and its importance and quality’ leads to a typology with four ideal types of business formats that vary with regard to the preceding process dynamics, and thus vary in their effects (see Table 4).

**Table 4:** **Typology of support and control in business formats in franchising in healthcare**

**Process dynamics**	**Types of business format**			
	**Extensive business format** ***(high control, much support of high importance or quality)***	**Supporting business format** ***(low control, much support of high importance or quality)***	**Bureaucratic business format** ***(high control, little support of low importance or quality)***	**Minimal business format** ***(low control, little support of low importance or quality)***
Level of overhead (sys)	High	Medium	Medium	Low
Level of bureaucracy (sys/unit)	High	Low	High	Low
Ease of system-wide adaptation (sys)	High	Medium	Medium	Low
Uniform presentation (sys/unit)	High	Medium	Medium	Low
Predictable/guaranteed performance levels (sys/unit)	High	Medium	Medium	Low
Accelerating implementation of practices (unit)	Medium	Medium	Low	Low
Resistance to change (clash autonomy) (unit)	High	Low	High	Low
Ease of local adaptation (unit)	Low	High	Low	High
Ease of knowledge sharing (unit)	High	Medium	Medium	Low

An *extensive business format* has high levels of support of high importance and quality, and units are heavily controlled. When used in the appropriate situation, this format helps to achieve results like efficiency, competitive advantages, and quality of care. It does so by making knowledge sharing easy, reducing the time unit actors spend on identifying appropriate work methods, and steering the unit actors’ behavior toward a uniform brand image, predictable performance, and implementation of system-wide changes. It is a suitable format for purchasers that desire geographically dispersed, uniform services and for unit actors that accept losing control and following the guidance of the formula. However, this format can lead to negative effects where professionals desire involvement and autonomy (“not invented by me”) and where units must heavily adapt to the needs of their localities. This format can also lead to inefficiencies through the overhead and bureaucracy required. Contractual payments can be relatively high to be reasonable.

A *supporting business format* combines substantial support of high perceived importance and quality with low control. This format is particularly valuable where clients and purchasers need customized care and where professionals seek autonomy in local implementation and adaptation in their own context, while feeling unburdened by support services. The actual autonomy and ownership feeling are lower when the support services are directed at both care and non-care related activities. It is a less efficient format when many decisions could be made on the system level, rather than requiring participation, and when system-wide adaptation is so important in the healthcare market that the franchisor must exert substantial effort into persuading unit actors. System 2 shares characteristics with this format.

A *bureaucratic business format* is characterized by ample control and little support of low perceived importance or quality. This format can have various negative effects in most types of healthcare services as it gives little room for local adaption and professionals’ ideas, as well as causing unit actors to feel unsupported. This format can, however, potentially lead to positive results in environments where standardized, lower cost healthcare services are preferred by clients and purchasers and where customization is not important, but overall seems sub-optimal in healthcare. System 1 used a business format in between the bureaucratic and extensive business format.

A *minimal business format* has low control and low support levels. This combination is suitable to satisfy clients and purchasers who seek customized care and/or when services are complex and professionalized, when professional associations play an important part in developing standards, or when unit actors desire autonomy and an ability to adapt locally. However, the low control and support levels are not very helpful for yielding substantial benefits from the franchise cooperation in regard to using suitable work methods and a uniform brand. Contractual payments should be relatively low in order to be reasonable. The format used by system 3 sits in between the extensive and minimal business format.

**Figure 1:**
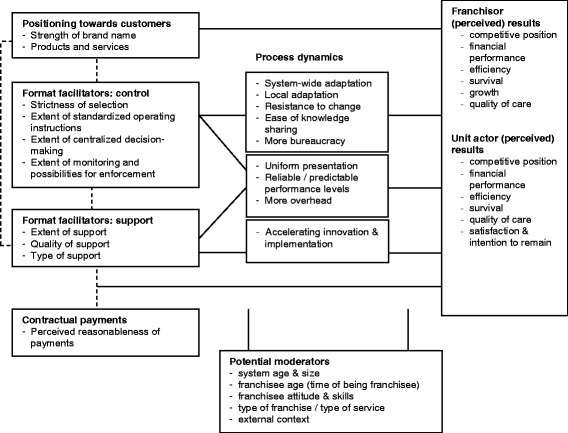
**Proposed model of the relationship among the business format, contractual payments and the results achieved within franchises to be tested in future research.**

As also follows from the discussion above, none of the business format designs seem favorable or unfavorable in all situations. The perceived effects of the same format can diverge across unit actors differing in attitudes, skills and length of stay in the system. Different external contexts (e.g., competitiveness, whether purchasers desire uniformity and predictability), positioning manners, and types of service require different control and support levels, as do franchises that work with existing organizations (fractional model) versus franchises with stand-alone units. Franchisees evaluate the contribution of support to results in relation to the fees they pay, as does the franchisor for the support he can deliver in relation to the fees received. We have mapped all these findings in a new model (Figure 1) depicting how combinations of business format elements are expected to relate to results via multiple intermediating processes and how age, size, attitude, skills, type of franchise, context, and type of service seem to moderate these relationships. This model requires further research.

Our study shows that the franchise literature originating from other sectors can provide valuable insights to healthcare scholars and practitioners. However, there are also differences that require a specific approach in healthcare. First, strong branding seems predominantly stimulating through the strong position that it provides in relation to stakeholders like insurers and the government (less so by attracting clients). Second, we found that unit actors do not consistently feel extensive support to be stimulating. Some do not want extensive support, as they want to bring in their own ideas and experiment in their localities, rather than risking central support that may not be applicable. Extensive support is sometimes even perceived as an infringement on professional autonomy, and people can feel that their own capacities and visions are not taken seriously. These risks can particularly appear where existing organizations become franchisees because actors receive the support and routines of the franchise in addition to those of their organization. Third, although some standardization can indeed prevent actors from opportunistic activities [38], an appropriate balance between high and low standardization seems beneficial to gain the advantages of standardization (achieving desired performance, acting as one system, sharing knowledge, deriving efficiency) on the one hand, and the advantages of freedom (delivering customized care on a local level with autonomy) on the other hand. For similar reasons, our study suggested that a balance between centralized and decentralized decision-making has positive effects for all. When a level of standardization or centralized decision-making is used that is inappropriate to the local situation of units and ideas of professionals, it results in waste because of resistance or threat to leave the system. Finally, we observed that franchises with business formats that are valued by customers and unit actors can still encounter difficulties because of the involvement of multiple external stakeholders (e.g., changing governmental policies, budget cuts).

### Limitations and directions for future research

The study also has some limitations that provide directions for future research. First, we lacked suitable data to quantitatively investigate results and to quantitatively support the qualitatively experienced, analyzed and interpreted relationships between results and business format elements. Therefore, we recommend large-scale quantitative tests of the developed model (see Figure 1) in a longitudinal design. Ideally, such research includes a baseline qualitative and quantitative study and one or more follow-up quantitative measurements in franchise systems that have planned to change a particular business format design element. Interviews in between the measurements should identify any affecting concurrent developments. Alternatively, a similar design could start with identifying typically low-, medium- and high-scoring franchise systems on a particular business format design element. Second, the study investigated the perceived impact of the business format design on client-related results as perceived by unit actors and franchisors, rather than as perceived by clients themselves. Scholars should investigate the relationship between the business format design and customer-related results (e.g., satisfaction, medical condition, costs) at the level of the customers themselves to really reveal what is the most valuable design to them (clients, insurers, referring providers). Third, the generalizability of the results of this in-depth qualitative study of three Dutch franchises is uncertain. Particularly in for-profit environments and low- and middle income countries, the motives of actors for franchising and thus the perceptions about the most desirable business formats to enhance the chance of success, may differ. Therefore, similar studies in other contexts are required. Finally, although we could draw our conclusions based on significantly diverging perceptions identified by within and between-case comparisons, it is possible that comparison with franchisors and unit actors that ‘failed’ or ceased franchising would have led to additional and fine-tuned insights in the choices that should not be made to enlarge the chance of success. Therefore, further research should compare the perceptions of operational franchisors and unit actors with failed/ceased ones.

## Conclusions

This study suggests that practitioners that use healthcare franchising as a model to achieve positive client-related, professional, strategic and/or organizational results need to carefully design their business format to increase the likelihood of actually achieving positive results. Franchisors seem to be able to stimulate results for all stakeholders if the positioning component comprises products and services that are valued by customers and (potential) unit actors and a strong brand name. They should try to avoid both extensively high and low levels of support and control to units, choosing instead the optimal level that partially diverges across systems, contexts, and unit actors. The processes of system-wide adaptation, local adaptation, knowledge sharing, predictable performance, uniform presentation, accelerated innovation and implementation, bureaucracy, level of overhead, and resistance to change explain why certain levels of support and control are related to results in different situations. It seems important to attune the type and amount of support and the level of control to the type of the service (e.g., desirable level of customization and professionalization), the external context, the franchise type, the unit actors’ skills, attitudes and years of working in the system, and the prioritization of goals (e.g., is growth and efficiency prioritized over quality and work satisfaction). Potential purchasers should determine whether the characteristics of the business format fit to their desires. The preceding conclusions are summarized in a typology and model that can be used as a starting point for practitioners and as a basis for future scholarly research. Further research is needed in other contexts like for-profit environments and low- and middle income countries to determine the generalizability of our findings.

## Endnotes

^a^http://www.entrepreneur.com/franchises/healthcare/indexhlth.html, http://www.bison.com/Healthcare_Franchises, http://www.franchisedirect.com/healthcareseniorcarefranchises/15 retrieved 24 May 2013

^b^http://www.franchisesales.co.uk/search/care-services-franchise-health-care-franchises, http://www.franchisedirect.co.uk/carefranchises/175, retrieved 24 May 2013

^c^http://canada.franchisesales.com/search/health-care-franchise-2, http://www.franchisedirectcanada.com/healthcare-senior-care-franchises-0505/, http://canada.franchisesales.com/search/care-franchise 24 May 2013

^d^http://www.erasmusmc.nl/commissies-cs/metc-cs/573270/reikwijdte [in Dutch].

## Additional file

Additional file 1: Table S1. Sources of data.
