# Supplementary material for: Creating advantages through franchising in healthcare: a qualitative, multiple embedded case study on the role of the business format
Source: BMC Health Serv Res. 2014 Nov 2;14:485. doi: 10.1186/s12913-014-0485-5 (PMC4226876; doi:10.1186/s12913-014-0485-5)
Supplement: Additional file 1: Table S1. — Sources of data. [file 12913_2014_485_MOESM1_ESM.doc]

Additional file 1 Table S1: Sources of data.

| System | No. of interviews | | | | | Archival documents | | Observations | |
| --- | --- | --- | --- | --- | --- | --- | --- | --- | --- |
|  | Franchisor representatives | Franchisee unit representatives | Company-owned unit representatives | Professionals in unit | Total number* | Number | Examples | Number | Examples |
| 1: Mental healthcare | 4 | 27 (of 4 different franchisees) | n.a. | 13 | 30 | 50+ | Business plan, internet forum, internal memo, minutes, media articles, operation manual, franchise contract | 4 | Care program council meeting, care program council conference, board meeting |
| 2: Eye-care | 15 | 20 (of 3 different franchisee units) | 13 (of 2 different units) | 11 | 48 | 50+ | Year report, minutes, media articles, internal memo, intranet, operation manual, franchise contract | 8 | Network days, franchisee-franchisor meeting, franchisor team meeting |
| 3: Intellectually disabled care | 5 | 8 (of 8 different franchisee units) | 1 | 13 | 18 | 22 | Year report, media articles, intranet, operation manual, franchise contract, quality and complaints year report | 4 | Franchise council meeting, site visits |

*total is lower than the sum of the columns, as some count double (e.g., some professionals are also franchisees: they were classified according to their stakeholder perspective).
